# Analysis of hematological parameters in rheumatoid arthritis patients receiving biological therapy: contribution to prevention of avoidable hematological complications

**DOI:** 10.17179/excli2022-4702

**Published:** 2022-03-08

**Authors:** Jana Pereckova, Silvia Martiniakova, Juraj Payer, Martin Falk, Zdenko Killinger, Tomas Perecko

**Affiliations:** 1 Center of Experimental Medicine, Slovak Academy of Sciences, Institute of Experimental Pharmacology and Toxicology, Bratislava, Slovak Republic; 2 Department of Cell Biology and Radiobiology, Institute of Biophysics of the Czech Academy of Sciences, v. v. i., Brno, Czech Republic; 3 Department of Food Technology, Faculty of Chemical and Food Technology, Slovak University of Technology, Bratislava, Slovak Republic; 4 Comenius University Faculty of Medicine, 5th Department of Internal Medicine, University Hospital Bratislava, Bratislava, Slovak Republic

**Keywords:** biological therapy, rheumatoid arthritis, IL-6, TNFalpha, hematologic parameters

## Abstract

Administration of biological therapy (BT) in rheumatoid arthritis (RA) patients is often associated with hematological complications, which result in switching among therapies. Thus, there is an instant need for suitable screening parameters that will help to individualize the therapy and minimize the onset of adverse effects. We analyzed the hematological profile of 99 RA patients receiving TNFα (Adalimumab - ADA, Golimumab - GOL, Etanercept - ETA) or IL-6 receptor (Tocilizumab - TCZ) inhibitors in order to find possible indicators to improve personalization of RA therapy. BTs significantly affect the levels of observed hematological parameters. In contrast to TNF-α inhibitors, TCZ normalized almost all monitored hematological parameters to values of healthy donors. Only GOL from the TNF-α inhibitors studied, was able to normalize neutrophil counts, as well as platelet indicators. Importantly, effects on the blood parameters (e.g. lymphocytes or platelet count) differ even within the same therapeutic group (anti-TNFα). Variable effects of individual biological agents in RA treatment point to importance to evaluate the patient's hematological profile to improve the selection of suitable BT. It will help to personalize the administration of BT and prevent unnecessary switching from an effective therapy just because of provocation of avoidable hematological complications.

## Introduction

Rheumatoid arthritis (RA) affects about 1 % of adults worldwide (4 times more frequent in women than in men) (Rudan et al., 2015[32]). As in other chronic inflammatory diseases, the onset and progression of RA are associated with the excessive production of inflammatory mediators by resident and/or infiltrated cells. Among the primary mediators involved in joint damage are pro-inflammatory cytokines, including tumor necrosis factor alpha (TNFα) and interleukin (IL)-6 (Conte Fde et al., 2008[13]). Both have proved to play an essential role in the development of RA especially via the induction of immune cells (Bellucci et al., 2016[5]; Tanaka, 2016[40]). Importantly, TNFα and IL-6 are also significant hematopoietic regulators. IL-6 has been implicated as a critical activator of myelopoiesis in response to chronic inflammation (Ishihara and Hirano, 2002[21]) and suppressor of lymphopoiesis (Maeda et al., 2005[23]), making IL-6 a key regulator of the lymphocyte/myelocyte balance. Similar alterations in hematopoiesis occur in clinical situations in which IL-6 is elevated, such as autoimmune diseases, acute infection, or in sepsis (Maeda et al., 2005[23]). TNFα is described as a bifunctional regulator of hematopoiesis. Its acute short-term upregulation stimulates the growth of immature immune cells; however, chronic long-term exposure induces a decrease of early myeloid progenitors resulting in neutrophilia and lymphopenia (Tanaka et al., 2010[39]; Ulich et al., 1989[43]). 

The treatment of RA patients is primarily focused on the anti-inflammatory effect (Smolen et al., 2017[36]). Most of the patients are treated with disease modifying anti-rheumatic drugs (DMARDs). The effect of conventional synthetic DMARDs (cs-DMARDs, e.g. methotrexate) impede or halt the inflammation but the induction of remission is low. Hence, most of the patients are forced to switch to biological therapy (BT), which targets specific soluble or cell-surface molecules of interest (Moots and Naisbett-Groet, 2012[25]; Smolen et al., 2017[36]). So far, several biological therapies (BTs) against different molecules (e.g., TNFα, IL-6 receptor (IL-6R)) have been used in RA clinical practice. The most frequent BTs antagonize TNFα (Golimumab- GOL; Adalimumab- ADA; Etanercept- ETA) and IL-6R (Tocilizumab, TCZ) (Hashimoto et al., 2014[19]; Orlewska et al., 2011[28]), respectively. Although a clear positive effect of anti-TNFα and anti-IL-6R treatment of RA has been demonstrated in a number of clinical trials (Nam et al., 2017[26]), the therapeutic intervention into the significant hematopoietic pathways regulated by TNFα or IL-6 can result in serious hematological abnormalities. In the case of anti-TNFα therapy, e.g. thrombocytopenia, neutropenia, aplastic anemia, and eosinophilia have been described (Bessissow et al., 2012[6]). The treatment with anti-IL6R therapy is associated with the neutropenia and higher hemoglobin level (Hashimoto et al., 2014[19]; Negrei et al., 2016[27]). 

While the complete blood cell count is regularly monitored before and with each administration of BTs, personalized selection of suitable BT with respect to the patient's hematological profile is still insufficient. Recent literature has evaluated and compared therapeutic and adverse effects of different anti-TNFα biologicals as if they form one therapeutically homogenous group (Aaltonen et al., 2012[1]; Choy et al., 2017[12]; Michaud et al., 2014[24]; Singh et al., 2009[34], 2011[35]). Such a general categorization may prevent more precise personalized therapy. Sing et al. and Michaud et al. showed that anti-TNFα biologics viewing as a group were associated with higher rate of total adverse effects and risk of discontinuation due to adverse effects, respectively (Michaud et al., 2014[24]; Singh et al., 2011[35]). According to Michaud et al. (2014[24]) anti-TNFα biologicals show similar efficacy in RA, thus their safety profile is an important determinant for decision making in RA treatment. Therefore, there is an urgent need for evaluating the comparative safety of different biologicals. 

Motivated by this unsatisfactory situation, we analyzed the hematological effects of different BT agents belonging to the group of TNFα inhibitors and compared them mutually as well as to the effects of an IL-6 receptor inhibitor, non-BT treatment, and healthy donors.

## Materials and Methods

### Participants

This study was conducted in accordance with the Declaration of Helsinki. Informed consent was obtained from all individual participants included in the study. The study was approved by the Ethics Committee of the University Hospital Bratislava (EK/29/2012, date: 15. 02. 2012). The study was characterized as a research project, and the donor identity was not registered. Patients were diagnosed according to the ACR/EULAR 2010 criteria for RA (Smolen et al., 2017[36]). The patients who had received disease modifying anti-rheumatic drugs (DMARDs) at the University Hospital Bratislava - Center for Biological Therapy in Rheumatology from January 2013 to May 2017 were targeted for inclusion in the present study. Disease activity was determined by 28-joint disease activity score (DAS28). Patients were excluded if they had any of the following conditions: 1) malignant diseases, 2) history of receiving a blood transfusion during the past three months, 3) acute inflammation or other infections, 4) chronic liver disease. The following demographic and clinical characteristics obtained from hospital file records were assessed at the time of recruitment: age, gender, duration of therapy, treatment (Table 1(Tab. 1)). All patients who started treatment with BT at the Center for Biological Therapy in Rheumatology were treated with standard or recommended dosing and routes of administration (s.c.).

### Blood sampling and hematological variables

All phlebotomies were performed in the morning and the donors were instructed to eat only a light meal before sampling. Blood (total volume of 18 ml) was collected directly onto buffered sodium citrate solutions, mixed immediately after the blood was drawn and processed within 2 hours. Hematological variables were analyzed with the ABX Pentra 60 hematological analyzer (Horiba Medical, Irvine, CA, USA). Neutrophil/lymphocyte ratio (NLR) and platelet/lymphocyte ratio (PLR) were calculated.

### Group definitions

Healthy donors (HD, n=20) were set as control. RA patients were divided into 2 main groups: without biological therapy (RA-DMARDs, n=20) and with biological therapy (RA-BT, n=79). The distribution of men/women in the groups corresponds to the occurrence of RA in the population. Out of the 99 RA patients in this study, RA patients receiving cs-DMARDs with DAS28>2.6 (not achieving remission) and RA-BT patients meeting the following criteria were included: DAS28>5.1 for RA activity despite the treatment with adequate doses of conventional synthetic DMARDs including methotrexate (MTX) for a minimum of 3 months, and no or moderate clinical response, according to the EULAR improvement criteria. At study entry, the patients continued their treatment including MTX and were concomitantly treated with BT. In RA-BT the following treatment groups were defined: TNFα blockers (GOL (n=20); ADA (n=20); ETA (n=19)) and IL-6R blocker (TCZ (n=20)).

### ESR and CRP

The erythrocyte sedimentation rate (ESR) was measured immediately after blood collection. C-reactive protein (CRP) levels were measured by immunoturbidimetric assay.

### Statistical analysis

A statistical power analysis was performed for sample size estimation. For the purpose of this study, comparisons between different pairs of two independent groups were planned. The effect size was 0.8, considered to be large enough using Cohen's (1988) criteria. With an alpha = 0.05 and power = 0.80, the projected sample size needed for this effect size is approximately N = 42 for the comparison between groups, with N2/N1 = 1 (calculated using G*Power 3.1.9.7 software, (Faul et al., 2007[16])). The number of available patients for this study was N = 39 - 40 (for each pair of tested groups), so it closely approached the required sample size and was adequate for the main objective of this study.

Values are shown as boxplots expressing medians (solid line), means (+), interquartile intervals Q1 -Q3 (Tukey´s); maxima and minima (whiskers) and outliers. Comparisons between selected groups were assessed with unpaired two-tailed Student´s t-test by using GraphPad Prism version 9.1.0 for Windows, GraphPad Software, San Diego, California USA, www.graphpad.com. P values less than 0.05 were considered as statistically significant. The correlations between DAS28 and selected BTs were calculated with the Pearson correlation coefficient.

## Results

### WBC counts are higher in anti-TNFα biologics

**Figure 1:**
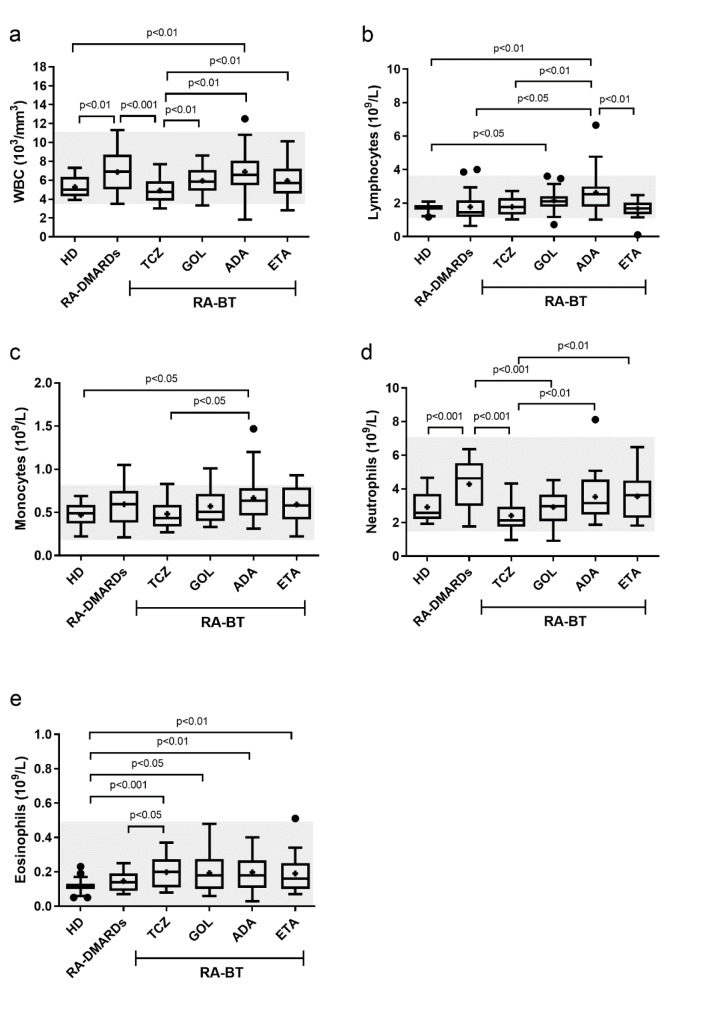
The number of peripheral immune cells in healthy donors (control) and RA patients undergoing a standard RA therapy (RA-DMARDs) or different types of biological treatment (RA-BT). The numbers of total white blood cells (WBC; a), lymphocytes (b), monocytes (c), neutrophils (d) and eosinophils (e) were compared for healthy donors (HD, n = 20), patients with RA without BT (RA-DMARDs; n = 20), and RA patients receiving distinct BT (RA-BT) - tocilizumab (TCZ, n = 20); golimumab (GOL, n = 20); adalimumab (ADA, n = 20); or etanercept (ETA, n = 19). Data are presented as box plots displaying the median, mean (+), 25^th^ and 75^th^ percentiles, minimum and maximum (whiskers), and outliers' values (●). The statistical difference was determined by unpaired two-tailed Student's t-test. The difference with p<0.05 was considered significant. The grey area indicates a normal reference range for healthy people.

First, the total level of white blood cells (WBC) was measured (Figure 1a(Fig. 1)). Compared to healthy donors, the WBC were significantly increased in RA patients receiving DMARDs (RA-DMARDs) and RA patients treated with ADA. Slightly elevated levels of WBC were also detected in RA patients treated with other TNF-α therapies (GOL and ETA). On the other hand, only the group treated with anti-IL6R therapy (TCZ) had a significantly lower amount of WBC compared to RA-DMARDs. The average levels of WBC in healthy donors (5.265±0.241×10^3^/mm^3^) and TCZ group (4.895±0.287×10^3^/mm^3^) were similar (Figure 1a(Fig. 1)).

Regarding mononuclear WBC, lymphocyte (LYM) and monocyte (MON) counts in RA-DMARDs were not significantly changed compared to healthy donors. However, three RA-DMARDs patients with lymphopenia were identified. LYM and MON numbers in RA patients receiving biological therapy (RA-BT), TCZ and ETA were not changed compared to healthy donors and RA-DMARDs. Nevertheless, compared to healthy donors and TCZ, RA patients treated with ADA had significantly higher numbers of both LYM and MON (Figure 1b, c(Fig. 1)). Interestingly, the LYM level in the ADA group was significantly increased even compared to RA-DMARDs (Figure 1b(Fig. 1)). On the other hand, in the GOL as well as the ETA group one patient with lymphopenia was recognized. Surprisingly, not only in the case of RA-DMARDs patients (4 cases), but in all RA-BT treatments, patients with monocytosis were identified (TCZ, GOL, ADA- 2 cases; ETA- 3 cases).

With the respect to polymorphonuclear leukocytes, RA-DMARDs had significantly higher numbers of neutrophils (NEU) when compared to healthy donors. Interestingly, TCZ and GOL, but not ADA and ETA, were able to significantly decrease the NEU counts in comparison with RA-DMARDs. However, this effect has been associated with the occurrence of neutropenia (15 % - TCZ and 5 % - GOL). The average level of NEU in TCZ-treated RA patients (2.415±0.2057×10^9^/L) was slightly, but not significantly lower when compared to healthy donors (2.914 ± 0.1930×10^9^/L). In comparison to TCZ, significant elevation of NEU counts was found in RA patients receiving ADA or ETA (Figure 1d(Fig. 1)). Interestingly, the numbers of eosinophils (EOS) in RA-BT were higher compared to RA-DMARDs and healthy donors. We identified peripheral eosinophilia in one patient treated with ETA (Figure 1e(Fig. 1)). Patients with GOL or ADA had significantly higher numbers of basophils (BAS) in comparison with healthy donors (data not shown).

### TNFα inhibitors decreased the level of red blood cells and hemoglobin but to a variable degree

**Figure 2:**
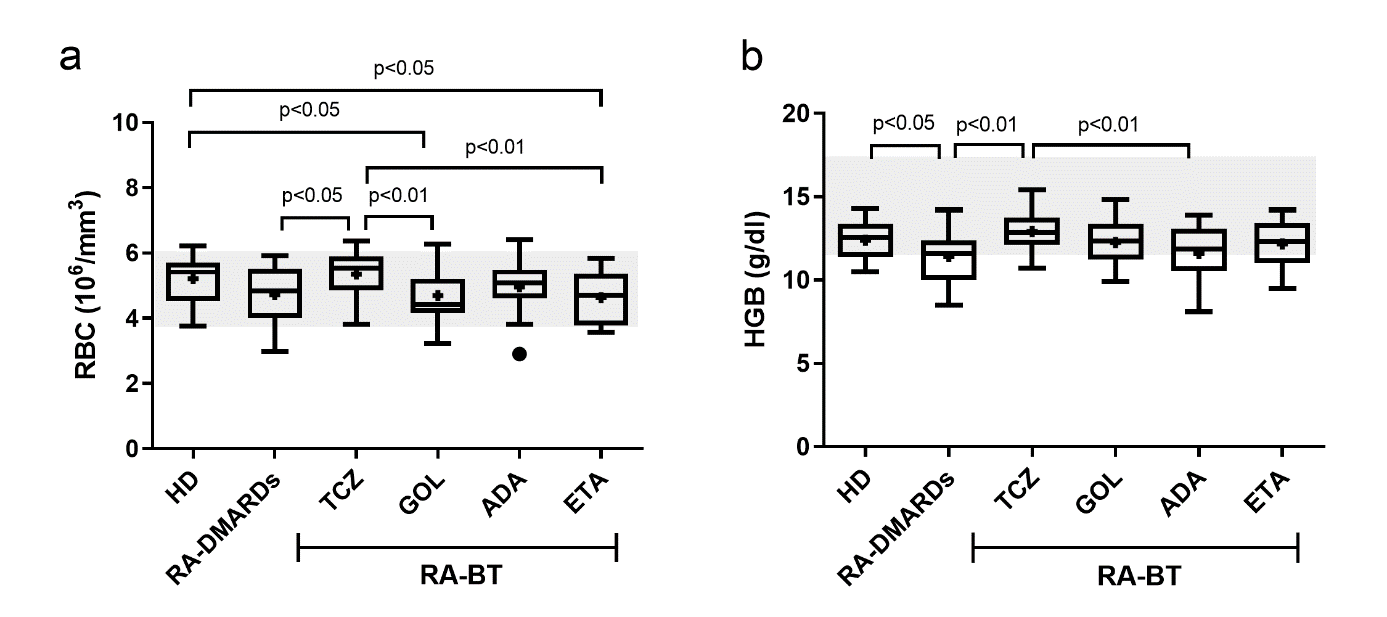
The number of red blood cells and hemoglobin levels in healthy donors (control), and RA patients undergoing a standard RA therapy (RA-DMARDs) or different types of biological treatment (RA-BT). The numbers of red blood cells (RBC; a) and hemoglobin levels (b) were compared for healthy donors (HD, n = 20), patients with RA without BT (RA-DMARDs; n = 20), and RA patients receiving distinct BT (RA-BT) - tocilizumab (TCZ, n = 20); golimumab (GOL, n = 20); adalimumab (ADA, n = 20); or etanercept (ETA, n = 19). Data are presented as box plots displaying the median, mean (+), 25^th^ and 75^th^ percentiles, minimum and maximum (whiskers), and outliers' values (●). The statistical difference was determined by unpaired two-tailed Student's t-test. The difference with p<0.05 was considered significant. The grey area indicates a normal reference range for healthy people.

Next, the red blood cells (RBC) and hemoglobin (HGB) levels were analyzed. Compared to healthy donors, there was a drop of RBC in RA-DMARDs (p=0.06) and three patients with anemia were identified. RA patients receiving TCZ had a significantly increased RBC number in comparison to RA-DMARDs and restored the RBC levels to values detected in healthy donors. In contrast, RA patients treated with GOL or ETA displayed significantly lower numbers of RBC relative to healthy donors and TCZ. Moreover, in all groups treated with different anti-TNFα therapy agents, patients with anemia were identified (GOL and ADA- 1 case; ETA- 2 cases) (Figure 2a(Fig. 2)).

Concerning HGB (Figure 2b(Fig. 2)), RA-DMARDs had significantly lower levels of HGB compared to healthy donors and TCZ group. Interestingly, a similar reduction of HGB level as observed in RA-DMARDs patients (11.43±0.364×g/dl) was detected in the ADA group (11.62±0.375×g/dl). GOL and ETA were able to slightly elevate HGB compared to ADA, but without a significant difference to other groups. Moderate anemia (8-10 g/dl) was identified in both RA-DMARDs (5 cases) and RA patients treated with anti-TNFα therapy (GOL- 1 case; ADA- 3 cases; ETA- 2 cases). As expected, low RBC levels corresponded to low HGB levels and vice versa (Figure 2(Fig. 2)). However, it is important to point out that also 53.3 % of healthy female donors suffered from mild (10-12 g/dl) anemia. Collectively, these results reveal that different types of TNFα inhibitors may have different effects on RBC counts and HGB levels. Specifically, while GOL and ETA significantly decreased RBC counts and relatively preserved HGB levels, the treatment with ADA had the opposite consequences.

### ADA reduces the elevated platelet values less than other anti-TNF-α treatments

**Figure 3:**
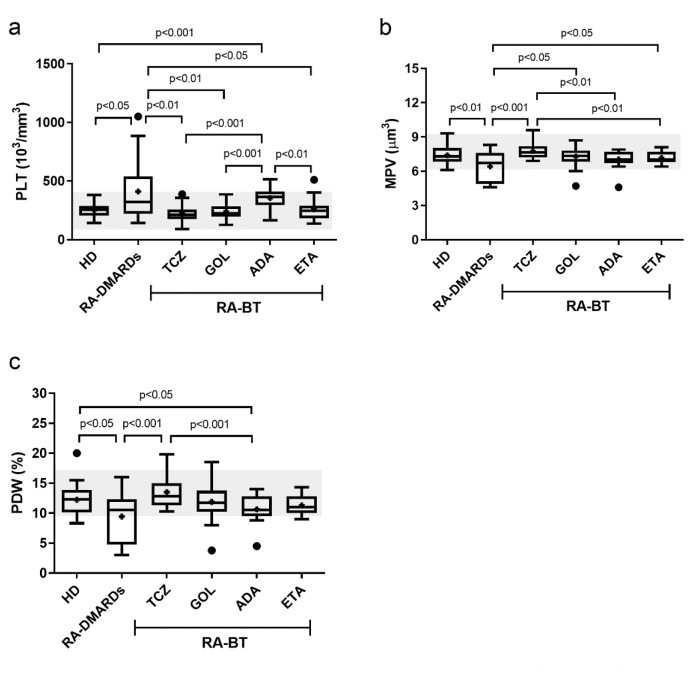
The number of platelets and values of mean platelet volume and platelet diameter width in healthy donors (control), and RA patients undergoing a standard RA therapy (RA-DMARDs) or different types of biological treatment (RA-BT). The numbers of platelets (PLT; a), mean platelet volume (MPV; b), and platelet diameter width (PDW; c) were compared for healthy donors (HD, n = 20), patients with RA without BT (RA-DMARDs; n = 20), and RA patients receiving distinct BT (RA-BT) - tocilizumab (TCZ, n = 20); golimumab (GOL, n = 20); adalimumab (ADA, n = 20); or etanercept (ETA, n = 19). Data are presented as box plots displaying the median, mean (+), 25^th^ and 75^th ^percentiles, minimum and maximum (whiskers), and outliers' values (●). The statistical difference was determined by unpaired two-tailed Student's t-test. The difference with p<0.05 was considered significant. The grey area indicates a normal reference range for healthy people.

The number of platelets (PLT), mean platelet volume (MPV) and platelet diameter width (PDW) were analyzed, too. A significant increase of PLT relative to healthy donors and all other treatment groups (excluding ADA), was detected in the RA-DMARDs group. In contrast, all BT agents except of ADA were able to effectively restore the normal PLT levels (Figure 3a(Fig. 3)). ADA, as the only TNFα inhibitor studied, was not able to decrease the PLT counts, to a comparable degree as observed for the BT treatment.

Opposing their high counts in RA-DMARDs patients, platelets in this group had significantly lower MPV than in other groups (healthy donors, TCZ, GOL, and ETA), except of ADA (Figure 3b(Fig. 3)). In line with these observations, also the average PDW was significantly higher in both healthy donors and TCZ groups in comparison to RA-DMARDs and ADA groups (Figure 3c(Fig. 3)). Interestingly, all the PLT levels, MPV, and PDW were similar for healthy donors (251.4×10^3^/mm^3^; 7.390 μm^3^; 12.25 %, respectively) and GOL (240.7×10^3^/mm^3^; 7.295 μm^3^; 11.90 %, respectively). RA patients treated with TCZ showed the lowest average level of PLT (223.1±16.3×10^3^/mm^3^) and highest average level of MPV (7.760±0.150 μm^3^) and PDW (13.51±0.588 %) compared to other groups (Figure 3(Fig. 3)). Hence, similar to the situation with RBC and HGB, ADA showed “the opposite” effects on platelets compared to the remaining two TNFα inhibitors studied (GOL and ETA).

**Figure 4:**
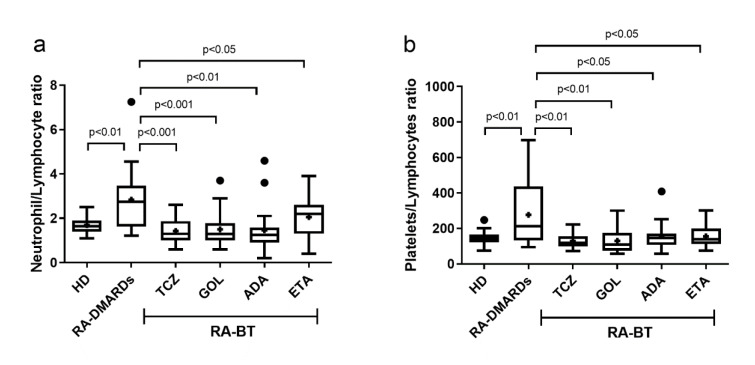
The neutrophils to lymphocytes ratio and platelets to lymphocytes ratio in healthy donors (control), and RA patients undergoing a standard RA therapy (RA-DMARDs) or different types of biological treatment (RA-BT). Differences in values of neutrophils to lymphocytes ratio (a) and platelets to lymphocytes ratio (b) were compared for healthy donors (HD, n = 20), patients with RA without BT (RA-DMARDs; n = 20), and RA patients receiving distinct BT (RA-BT) - tocilizumab (TCZ, n = 20); golimumab (GOL, n = 20); adalimumab (ADA, n = 20); or etanercept (ETA, n = 19). Data are presented as box plots displaying the median, mean (+), 25^th ^and 75^th^ percentiles, minimum and maximum (whiskers), and outliers' values (●). The statistical difference was determined by unpaired two-tailed Student's t-test. The difference with p<0.05 was considered significant.

The neutrophil/lymphocyte ratio (NLR) and platelet/lymphocyte ratio (PLR), the proposed indicators of the disease status, were calculated for healthy donors and RA±BT patients. As expected, RA-DMARDs had significantly higher NLR and PLR compared to healthy donors. Surprisingly, individual RA-BTs showed mutually comparable values of PLR, in spite of their different effects on the blood parameters (Figure 4(Fig. 4)).

### The majority of RA patients receiving TCZ showed remission

**Figure 5:**
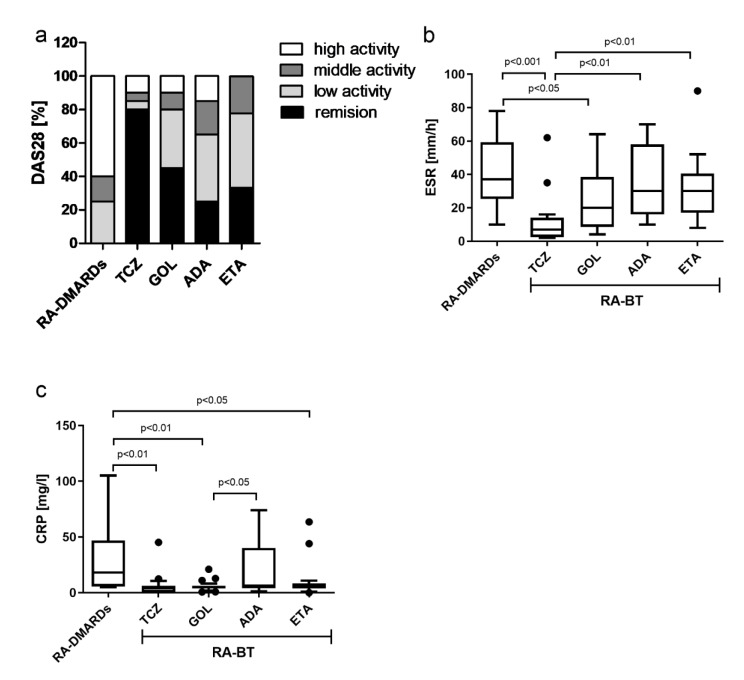
DAS, ESR and CRP parameters of tested subjects. a) The proportion of disease activity score (DAS28) indicating remission, low- middle- and high-activity of disease and RA patients undergoing a standard RA therapy (RA-DMARDs) or different types of biological treatment (RA-BT). The levels of erythrocyte sedimentation rate and C-reactive protein (CRP) in RA patients without BT (RA-DMARDs), and the effects of treatment with BT (RA-BT). Differences in values of erythrocyte sedimentation rate (ESR; b) and C-reactive protein (CRP; c) were compared for patients with RA without BT (RA-DMARDs; n = 20), and RA patients receiving distinct BT (RA-BT) - tocilizumab (TCZ, n = 20); golimumab (GOL, n = 20); adalimumab (ADA, n = 20); or etanercept (ETA, n = 19). Data are presented as box plots displaying the median, mean (+), 25^th^ and 75^th^ percentiles, minimum and maximum (whiskers), and outliers' values (●). The statistical difference was determined by unpaired two-tailed Student's t-test. The difference with p<0.05 was considered significant.

Finally, 28 joint disease activity score (DAS28), erythrocyte sedimentation (ESR), and C-reactive protein (CRP) parameters were analyzed. The treatments with different BTs significantly decreased the DAS28 values in comparison with the RA-DMARDs. Eighty percent of RA patients receiving TCZ showed remission according to the DAS28. There were 43.8 % more patients compared to GOL, 68.8 % more patients compared to ADA and 58.4 % more patients compared to ETA. In anti-TNFα treatment groups (GOL (35 %), ADA (40 %), and ETA (44.4 %)), comparably high numbers of patients with low activity of DAS28 (2.8-3.2) were detected in addition to patients in remission. Interestingly, only in ETA, no patients with high activity of DAS28 (> 5.1) were detected (Figure 5a(Fig. 5)). In line with these observations, the treatment with TCZ significantly lowered also the ESR and CRP levels in comparison to the RA-DMARDs therapy. Similar patterns were found only for GOL from the anti-TNFα remedy group. Patients receiving ADA did not show significant changes in both ESR and CRP. ETA then significantly decreased only the CRP levels compared to RA-DMARDs (Figure 5b, c(Fig. 5)). Taking together, only GOL from the group of TNFα inhibitors, and TCZ (IL-6R inhibitor), were able to significantly reduce both ESR and CRP levels compared to RA-DMARDs.

See also the Supplementary Information.

## Discussion

Several studies linked the presence of hematological disorders with RA disease activity, treatment prognosis, the onset of additional hematological abnormalities, or with conventional synthetic disease-modifying anti-rheumatic drugs (DMARDs) co-treatment (Autrel-Moignet and Lamy, 2014[2]; Azuma et al., 2017[3]; Bessissow et al., 2012[6]; Boilard et al., 2012[7]; Hensel et al., 2017[20]). The results presented here have shown that BT may contribute to disruption of hematological homeostasis, which could be followed by the onset of side effects and lead to therapy switch. Importantly, effects of individual TNFα and IL-6R inhibitors showed compound-specific effects on the RA patients' hematological profile. Hence, consideration of individual anti-TNFα therapeutics as one therapeutically homogenous rating group may cause a misinterpretation of the therapeutic effects and/or occurrence of hematological abnormalities, thereby precluding personalized therapy planning and increasing risk of its failure. Our results thus emphasize the importance of evaluating RA patient's hematological profile before the selection of BT. While more patients must be studied to delineate some clinically applicable guidelines for monitoring of therapy efficiency and safety, some relationships between studied BTs and their effects on blood parameters may be delineated, as discussed in the following paragraphs. 

**Figure 6:**
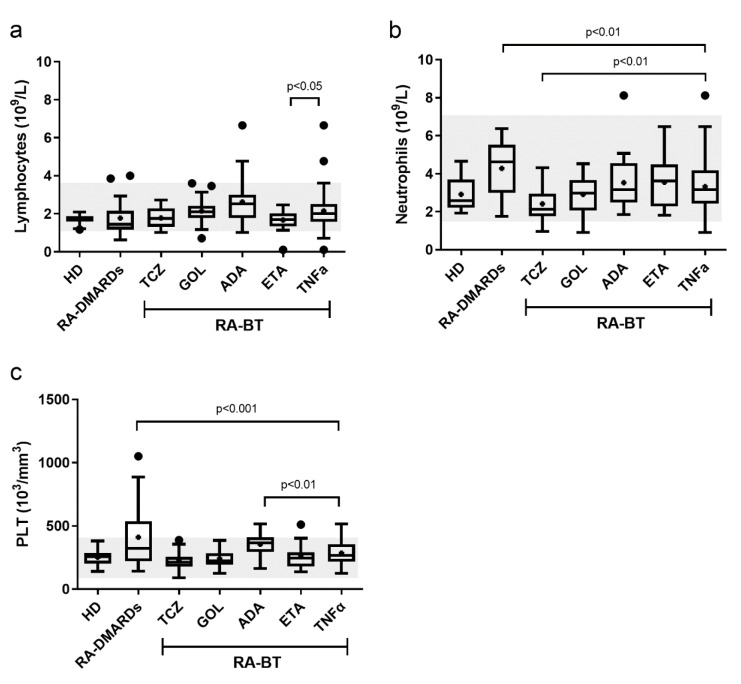
Different significance when comparing individual BTs and anti-TNFα as one group in (a) lymphocytes, (b) neutrophils or (c) platelets (PLT) numbers. Compare with Figures 1b, 1d and 3a, respectively. Healthy donors (HD), patients with RA without BT (RA-DMARDs; n = 20), and RA patients receiving distinct BT (RA-BT) - tocilizumab (TCZ, n = 20); golimumab (GOL, n = 20); adalimumab (ADA, n = 20); or etanercept (ETA, n = 19) were compared. Data are presented as box plots displaying the median, mean (+), 25^th^ and 75^th^ percentiles, minimum and maximum (whiskers), and outliers' values (●). The statistical difference to anti-TNFα group was determined by unpaired two-tailed Student's t-test. The difference with p<0.05 was considered significant. The grey area indicates a normal reference range for healthy people. For readability reasons, only significance for TNFα group are indicated. Significance between other groups are shown in Figures 1b, 1d and 3a, respectively.

Most studies have suggested that the reduction of RA-associated inflammation due to the administration of BTs is accompanied by a decrease of the WBC (compared to the active disease) (Bessissow et al., 2012[6]; Rigby et al., 2017[31]; Sag et al., 2018[33]; Syed and Pinals, 1996[38]). However, we have discovered that some TNFα inhibitors studied in the present study, such as ADA, may keep the total WBC count significantly above the level in healthy donors. In addition, we detected a higher level of the total lymphocyte counts in GOL and ADA groups. This might be related to the fact that TNFα has a bi-functional role in the growth of hematopoietic stem and progenitor cells and its inhibition is associated with the onset of lymphoproliferative disorders and pathological abnormalities (Autrel-Moignet and Lamy, 2014[2]; Bessissow et al., 2012[6]; Picchianti Diamanti et al., 201[30]; Tian et al., 2014[42]). Interestingly, while the therapeutically mediated decrease of the lymphocyte counts is generally correlated to RA activity, ADA and GOL treatments improved the DAS28 score even in presence of higher lymphocyte levels. Moreover, merging of all three anti-TNFα BTs together may mask the lower numbers of WBC or LYM generated by ETA and diminishing the higher LYM number provoked by ADA (Figure 6a(Fig. 6)). In agreement with recent studies (Hensel et al., 2017[20]), we observed monocytosis in both RA-DMARDs and RA-BT groups. Chara et al. suggested that the absolute number of monocytes could have a predictive value in terms of clinical response to ADA (Chara et al., 2012[10]). We did not observe correlation between the patient's DAS28 and monocyte level in the case of monocytosis (data not shown). 

In contrast to lymphocytes, the neutrophil levels are increased in chronic inflammatory diseases, such as RA (Autrel-Moignet and Lamy, 2014[2]). Enhanced neutrophil numbers may thus in part explain the elevation of total WBC. It is known that both anti-TNFα and anti-IL6R therapies decrease the neutrophil numbers, with the anti-IL6R therapy (TCZ) leading more frequently to neutropenia than the anti-TNFα (e.g., ETA or ADA) therapy (Espinoza et al., 2017[15]; Gaber et al., 2016[18]; Wright et al., 2014[45]). In the present study, TCZ decreased the neutrophil numbers even below the levels found in healthy donors, causing neutropenia in three patients. The pathophysiological mechanism behind this phenomenon remains unclear (Autrel-Moignet and Lamy, 2014[2]). In addition, one patient with neutropenia was identified in the GOL group. However, in this case, neutropenia was associated with leukopenia as well as with basophilia, which indicates the presence of infection, e.g., tuberculosis (Cantini et al., 2014[8]). Importantly, among anti-TNFα BTs in our study, GOL was the only agent capable of significantly lowering the neutrophil numbers in comparison with RA-DMARDs. Thus, the generalization of different TNFα inhibitors as one therapeutically homogeneous group lead to false conclusions regarding the neutrophil status (compare Figure 1d(Fig. 1) *vs.* Figure 6b(Fig. 6)).

Interestingly, eosinophils were elevated in RA-BT patients, no matter what anti-TNFα or anti-IL6R therapy had been administered. *In vivo *experiments of Chen et al. showed that eosinophil accumulation in the joint protects the bone from damage (Chen et al., 2016[11]). Thus, physiologically higher levels of eosinophils in blood seem to have a positive role in the treatment of RA. On the other hand, BT-induced eosinophilia, described also in our observation, was related with skin symptoms and hypersensitivity (Azuma et al., 2017[3]). 

Anemias are often present in patients with active RA and are associated with physical disabilities and increased mortality (Cavill et al., 2006[9]). It is in line with our observations of lower total RBC and HGB counts, which were associated with the identification of several anemic patients. Mechanisms involved are pathogenic iron homeostasis and/or impaired erythropoiesis (Weiss and Goodnough, 2005[44]). We showed decreased RBC numbers and a drop of HGB to a lower limit of the normal blood HGB concentration after the anti-TNFα treatment. This was further associated with the identification of anemic patients treated with different anti-TNFα BTs. Importantly, one case of a switch from an effective ADA therapy (DAS28 = 2.9) due to the onset of moderate anemia was recorded. On the other hand, based on the fact that IL-6 is essential in anemia related to chronic diseases, it is possible that anti-IL6R therapy could improve anemia markers more effectively than other biologics (Suzuki et al., 2017[37]). Similar to Paul et al. (2018[29]), our results show that TCZ but not anti-TNFα biologicals significantly increased the RBC and HGB levels. Within anti-TNFα group, the effects on RBC and HGB were agent-specific: the RBC levels in RA patients receiving GOL or ETA were significantly lower than in healthy donors or TCZ-treated patients.

It is well known that PLTs play an important role in the pathogenesis of RA (Boilard et al., 2012[7]) and in combination with ESR and CRP are important clinical parameters in the evaluation of disease activity and therapy effectiveness (Dahlqvist et al., 1988[14]). We found increased PLT numbers in RA without BT and identified thrombocytosis in several patients. On the other hand, thrombocytopenia is known to be associated with TNFα blockers (Bessissow et al., 2012[6]). In our study, ADA was the only BT that did not lower PLT numbers. This effect could have been overlooked in previous works (Choy et al., 2017[12]; Kihara et al., 2017[22]) which considered individual anti-TNFα BTs as one therapeutic group (compare Figure 3a(Fig. 3) *vs.* Figure 6c(Fig. 6)). However, no case of thrombocytopenia was observed in patients treated with anti-TNFα BTs. On the contrary, in both ADA and ETA, one case of thrombocytosis was identified. This is probably related to the moderate activity of the disease in both patients. Similar to GOL, TCZ significantly decreased PLT numbers to the level detected in healthy donors. Further, our results agree with observations of Bath and Butterworth (1996[4]) who recognized that high production of PLT is associated with a decrease of their volume (MPV) and diameter width (PDW), and vice versa. This makes both MPV and PDW promising markers of inflammation in chronic diseases (Yazici et al., 2010[46]). 

Many authors in recent studies suggest the neutrophil/lymphocyte ratio (NLR) and platelet/lymphocyte ratio (PLR) as markers of RA activity (Fu et al., 2015[17]; Tekeoglu et al., 2016[41]). However, we point to limitations of these markers as the indicators of the disease activity in patients treated with BTs. Apart from RA-DMARDs, the lymphocyte count is influenced by BT administration. For instance, TCZ, in contrast to other studied biologicals, normalized most of the hematological parameters to the levels similar to healthy donors. Also, most of patients who achieved clinical remission were treated with TCZ. However, when looking on the NLR and PLR ratios, TCZ had a similar effect compared to other biologicals. 

So far, some studies comparing the effectiveness of anti-IL6R and anti-TNFα BTs provided inconclusive results (Choy et al., 2017[12]; Kihara et al., 2017[22]). Based on our and other published data, we believe that one of the important sources of the disagreement poses in combining individual anti-TNFα therapies into one rating group (Choy et al., 2017[12]; Michaud et al., 2014[24]; Singh et al., 2011[35]). This may mask important differences among anti-TNFα biologics. In the present work, we showed that while the inhibition of IL-6R is associated with neutropenia and monocytosis, different anti-TNFα therapies cause a wide spectrum of changes in hematological variables. While more patients need to be analyzed in order to propose clinically applicable guidelines for RA treatment monitoring based on BT-specific hematological parameters, we have recognized here some agent-specific effects of anti-RA BTs, which could be possibly embedded into a monitoring protocol.

## Conclusions

In conclusion, BTs significantly affect the levels of observed hematological parameters. Importantly, the effects on the blood parameters differ even within the same therapeutic group (anti-TNFα). Although BTs in most cases keep the hematological variables within the physiological interval, in cases close to the pathological state, BTs may contribute to the development of serious hematological complications. Regarding the significant involvement of both main targets for biological treatment in RA - TNFα and IL-6 - in hematopoiesis regulation, our results emphasize the importance of the monitoring of the patient´s complete hematological profile during biological therapy. As we show the efficacy of anti-TNFα BTs is comparable, the safety profile of distinct remedy could be important for decision making for both the therapist and the patient. While the number of patients available for analysis in the present study does not allow the elaboration of clinically applicable guidelines for RA treatment monitoring based on BT-specific hematological parameters, we provide a matrix of data that may, after additional testing, significantly contribute to this effort and help to prevent switching from an effective therapy just due to avoidable hematological complications.

## Declaration

### Data availability

The data that supports the findings of this study are available from the Institute of Experimental Pharmacology and Toxicology but restrictions apply to the availability of these data, which is used under license for the current study at the University Hospital Bratislava, and so are not publicly available. Data is however available from the authors upon reasonable request and with the permission of the Institute of Experimental Pharmacology and Toxicology and the University Hospital Bratislava.

### Conflict of interest

The authors declare that they have no conflict of interest. 

### Author contributions

JP, JPa, ZK, TP designed the experiment; JP, SM, TP carried out the experiments; JP, SM, TP analyzed the data; JP, SM, TP wrote the paper. JPa, MF, ZK supervised the study, participated in the interpretation of the results as well as in the writing and critical reviewing of the manuscript. All authors read and approved the final manuscript.

### Acknowledgments

The authors would like to thank Viera Jancinova, Tatiana Macickova and Zuzana Strakova from the Institute of Experimental Pharmacology and Toxicology for technical support. We thank our dear co-worker and friend Michaela Pekarova for her exceptional support.

### Funding statement

The work was supported by the grant VEGA 2/0029/16 Ministry of Education, Science, Research and Sport of the Slovak Republic (JP, SM, JPa, ZK, TP) and by grants of the Czech Science Foundation (TP, GACR No. 17-08066Y), and Ministry of Education, Youth and Sports (MF, LTAUSA17160). JP was supported by Programme to support prospective human resources - post Ph.D. candidates (No. L200041802). The funders had no role in study design, data collection and analysis, decision to publish, or preparation of the manuscript.

## Supplementary Material

Supplementary information

## Figures and Tables

**Table 1:**
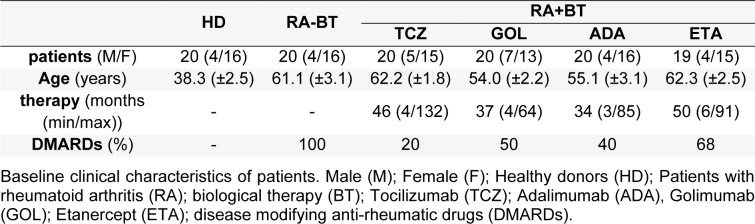
Baseline characteristics of patients and healthy donors
